# Coronary artery bypass grafting and perioperative stroke: imaging of atherosclerotic plaques in the ascending aorta with ungated high-pitch CT-angiography

**DOI:** 10.1038/s41598-020-70830-4

**Published:** 2020-08-17

**Authors:** Ulrika Asenbaum, Richard Nolz, Stefan B. Puchner, Tobias Schoster, Lukas Baumann, Julia Furtner, Daniel Zimpfer, Guenther Laufer, Christian Loewe, Sigrid E. Sandner

**Affiliations:** 1 Department of Biomedical Imaging and Image-Guided Therapy, Medical University of Vienna, Vienna General Hospital, Waehringer Guertel 18-20, 1090 Vienna, Austria; 2 Center of Medical Statistics, Informatics, and Intelligent Systems (CEMSIIS), Medical University of Vienna, Vienna, Austria; 3 Division of Cardiac Surgery, Medical University of Vienna, Vienna, Austria

**Keywords:** Cardiovascular diseases, Risk factors, Outcomes research

## Abstract

Perioperative stroke is a devastating complication after coronary artery bypass graft (CABG) surgery, with atherosclerosis of the ascending aorta as important risk factor. During surgical manipulation, detachment of plaques can lead to consecutive embolization into brain-supplying arteries. High-pitch computed tomography angiography (HP-CTA) represents a non-invasive imaging modality, which provides the opportunity for comprehensive imaging of the ascending aorta, including plaque detection and advanced characterization. In our present retrospective study on 719 individuals, who had undergone HP-CTA within 6 months prior to CABG, atherosclerotic disease of the ascending aorta was evaluated with respect to perioperative stroke rates. For image analysis, the ascending aorta was divided into a proximal and distal part, consisting of four segments, and evaluated for presence and distribution of calcified and mixed plaques. All patients with perioperative stroke presented with atherosclerotic disease of the ascending aorta. The stroke rate was significantly associated with the presence and extent of atherosclerotic disease. Patients burdened with mixed plaques presented with significantly higher perioperative stroke rates. This study demonstrates that HP-CTA allows accurate evaluation of plaque extent and composition in the ascending aorta, and therefore may improve risk stratification of stroke prior to CABG.

## Introduction

Perioperative stroke is a devastating complication after coronary artery bypass graft (CABG) surgery^1,2^, with atherosclerosis of the ascending aorta as important risk factor^3,4^. During surgical manipulation, detachment of plaques can lead to consecutive embolization into brain-supplying arteries^5,6^. As non-calcified plaque components are more susceptible to rupture and erosion^7,8^, plaque characterization is particularly important. Thus, the extent, distribution, and composition of plaques have an influence on surgical planning to prevent atheroembolic stroke^7,9,10^. Consequently, preoperative plaque imaging is highly desirable to allow for planned modifications of surgical technique to reduce or avoid manipulation of the aorta.

Second-, and third-generation dual-source scanners enable fast acquisition of datasets of the entire aorta, whereby image quality becomes less dependent on heart rate and pulsation artefacts. By means of imaging of the ascending aorta prior CABG, the elimination of pulsation artifacts may be considered to be the key requirement of CTA. In this context, ungated high-pitch protocols demonstrated excellent image quality even without premedication for heart rate control (e.g. beta-blockers) and at similar or even lower radiation dose compared with both gated, and slow-pitch gated protocols^11–14^. Additionally, this technique provides the opportunity for comprehensive imaging of the ascending aorta, including plaque detection and advanced characterization based on Hounsfield unit (HU) measurements of their components^15,16^.

The aim of this study was to assess the extent and composition of atherosclerotic plaques in the ascending aorta with ungated high-pitch computed tomography angiography (CTA), with respect to perioperative stroke rates after CABG.

## Materials and methods

### Study design

This was a single-center, retrospective, cross-sectional study of consecutive patients who underwent isolated CABG surgery between January 2009 and December 2016. This study was conducted in accordance with the declaration of Helsinki and was approved by the ethic committee of the Medical University Vienna (Ethikkommission der Medizinischen Universität Wien, 1090 Vienna, Austria) (No. 1239/2014). The IRB waived the need to obtain written, informed consent.

### Study population

There were 2,320 consecutive patients who underwent isolated CABG during the specified time period. Patients to whom the following criteria applied were excluded from analysis: (1) pre- and/or postoperative, extracorporeal membrane oxygenation (n = 54); and (2) no preoperative CTA imaging (n = 1,025). Based on the heterogeneity of the study group due to the usage of different scanner types, and several modifications of imaging protocols during the defined time period, we also excluded patients who underwent preoperative non high-pitch CTA (retrospective, and prospective gated, n = 522). Thus, 719 consecutive patients who underwent ungated high-pitch CTA within a median time interval of 11 days (IQR 3–42 days) prior to CABG were analyzed. A part of the study population was included in a previously described cohort^17^. Patient characteristics are given in Table 1.

**Table 1:** Patient characteristics.

	Overall (n = 719)	No perioperative stroke (n = 707)	Perioperative stroke (n = 12)	*p*
Sex, female	132/719 (18.4%)	126/707 (17.8%)	6/12 (50.0%)	0.012
Age, years	68.0 (60.3–74.4)	68.1 (60.9–74.3)	64.7 (61.9–72.8)	0.530
Height, cm	173.0 (168.0–178.0)	173.0 (168.0–178.0)	166.5 (160.5–174.5)	0.203
Weight, kg	82.0 (74.0–94.0)	82.0 (74.0–94.0)	79.0 (73.3–99.5)	0.698
BMI, kg/m^2^	28.0 (25.0–31.0)	28.0 (25.0–31.0)	28.0 (23.8–31.8)	0.993
Smoking history	339/719 (47.1%)	330/707 (46.7%)	9/12 (75.0%)	0.051
Active smokers	127/719 (17.7%)	124/707 (17.5%)	3/12 (25.0%)	0.453
Family history of coronary heart disease	117/719 (16.3%)	115/707 (16.3%)	2/12 (16.7%)	1.00
Diabetes mellitus	285/719 (39.7%)	278/707 (39.3%)	7/12 (58.3%)	0.207
IDDM	107/719 (14.9%)	104/707 (14.7%)	3/12 (25.0%)	
Dyslipidemia	565/719 (78.6%)	556/707 (78.6%)	9/12 (75.0%)	0.727
Renal insufficiency, mild to moderate	96/719 (13.4%)	91/707 (12.9%)	5/12 (41.7%)	0.014
Dialysis	14/719 (1.9%)	10/707 (1.4%)	4/12 (33.3%)	< 0.001
Serum creatinine, mg/dl	1.0 (0.85–1.2)	1.0 (0.85–1.2)	1.28 (0.83–3.05)	< 0.001
Hypertension	651/719 (90.5%)	641/707 (90.7%)	10/12 (83.3%)	0.316
Carotid stenosis and history of stroke	71/719 (9.9%)	69/707 (9.8%)	2/12 (16.7%)	0.336
Asymptomatic carotid stenosis	162/719 (22.5%)	158/707 (22.3%)	4/12 (33.3%)	0.483
PAOD	135/719 (18.8%)	131/707 (18.5%)	4/12 (33.3%)	0.253
Chronic lung disease	206/719 (28.7%)	202/707 (28.6%)	4/12 (33.3%)	0.552
Previous CABG	12/719 (1.7%)	11/707(1.6%)	1/12 (8.3%)	0.184
Previous heart valve operation	3/719 (0.4%)	3/707 (0.4%)	0/12	1.000
Previous myocardial infarction	378/719 (52.6%)	369/707 (52.2%)	9/12 (75.0%)	0.117
Ejection fraction, %	55.0 (45.0–60.0)	55.0 (45.0–60.0)	60.0 (45.0–60.0)	0.341
Atrial arrhythmia	95/719 (13.2%)	94/707 (13.3%)	1/12 (8.3%)	1.000
**Coronary artery disease**				
One-vessel	21/719 (2.9%)	21/707 (3.0%)	0/12	0.513
Two-vessel	85/719 (11.8%)	84/707 (11.9%)	1/12 (8.3%)	
Three-vessel	613/719 (85.3%)	602/707 (85.1%)	11/12 (91.7%)	
**Indication for surgery**				
Elective	457/719 (63.6%)	450/707 (63.6%)	7/12 (58.3%)	0.796
Urgent	234/719 (32.5%)	229/707 (32.4%)	5/12 (41.7%)	
Emergency	28/719 (3.9%)	28/707 (4.0%)	0/12	

cm, centimeter; kg, kilogram; BMI, body mass index; kg/m^2^, kilogram per square meter; NIDDM, non-insulin-dependent diabetes mellitus; IDDM, insulin-dependent diabetes mellitus; mg/dl, milligrams per deciliter; PAOD, peripheral artery occlusive disease, CABG, coronary artery bypass grafting, NYHA, New York Heart Association classification; p, no perioperative stroke versus perioperative stroke.

### Ungated high-pitch computed tomography angiography

All examinations were performed on a second-, or third-generation dual-source computed tomography system. Following scanners were used: Somatom Definition Flash (Siemens Healthineers; n = 602, 83.7%), Somatom Drive (Siemens Healthineers; n = 79, 11.0%), and Somatom Force (Siemens Healthineers; n = 38, 5.3%). The institutional standard protocol (Table 2) included a single acquisition of the entire aorta with a high pitch to reduce motion artifacts, particularly at the level of the ascending aorta. No premedication for heart rate control was applied. A double-head power injector (Injektron CT2, Medtron AG, Saarbrücken, GER) was used for intravenous injection of a non-ionic contrast medium (Iomeron 400, Bracco-Austria, Vienna, Austria), via antecubital vein, followed by a 40 ml saline chaser bolus. Examinations on the Somatom Definition Flash scanner were performed after injection of 40 ml Iomeron 400 at an injection rate of 2.5 ml/s. For the Somatom Drive and the Somatom Force scanner, amount of contrast medium, injection rate, and tube voltage were adjusted according to body weight. The individual radiation dose was estimated using the dose-length product (DLP) given by the CT system.

**Table 2:** Technical details of dual-source computer tomography angiography.

Acquisition parameters			
Scanner type	Somatom defintion flash^a^	Somatom drive^a^	Somatom force^a^
Tube voltage (refkV)	120	110	80
Tube current (refmAs, CD4D)	116	140	194
Collimation (mm)	128 × 0.6	128 × 0.6	192 × 0.6
Rotation time (s)	0.28	0.28	0.25
Pitch	2.4	2.4	1.9
Soft kernel	B31f.	I30f.	Bv40, Bv36
Iteration	None	Admire strength 3	Admire strength 3

Adjustment of contrast medium			
Amount of contrast medium, ml	Body weight, kg	Tube voltage, kV	Injection rate, ml/s
20	< 60	70–80	1.3
30	60–80	90–100	2
40	80–100	110–120	2.5

refkV, reference kilovolt; refmAs, reference milliampere seconds; mm, millimeter; sec, seconds; ml, milliliter; kg, kilogram; kV, kilovolt; ml/sec, milliliter/second.

^a^Siemens healthineers.

Post processing included maximum intensity projections and was performed from thin transversal image slices on a Multimodality Workplace (MMWP, Siemens Healthineers, Erlangen, GER) in the coronal and sagittal views, with a slice thickness of 3 mm and 1 mm, and a reconstruction increment of 2 mm and 0.8 mm, respectively. All CTA images were transferred to a picture archiving and communication system (IMPAX, Agfa Healthcare, Mortsel, BEL).

### Definitions and image interpretation

**Figure 1:**
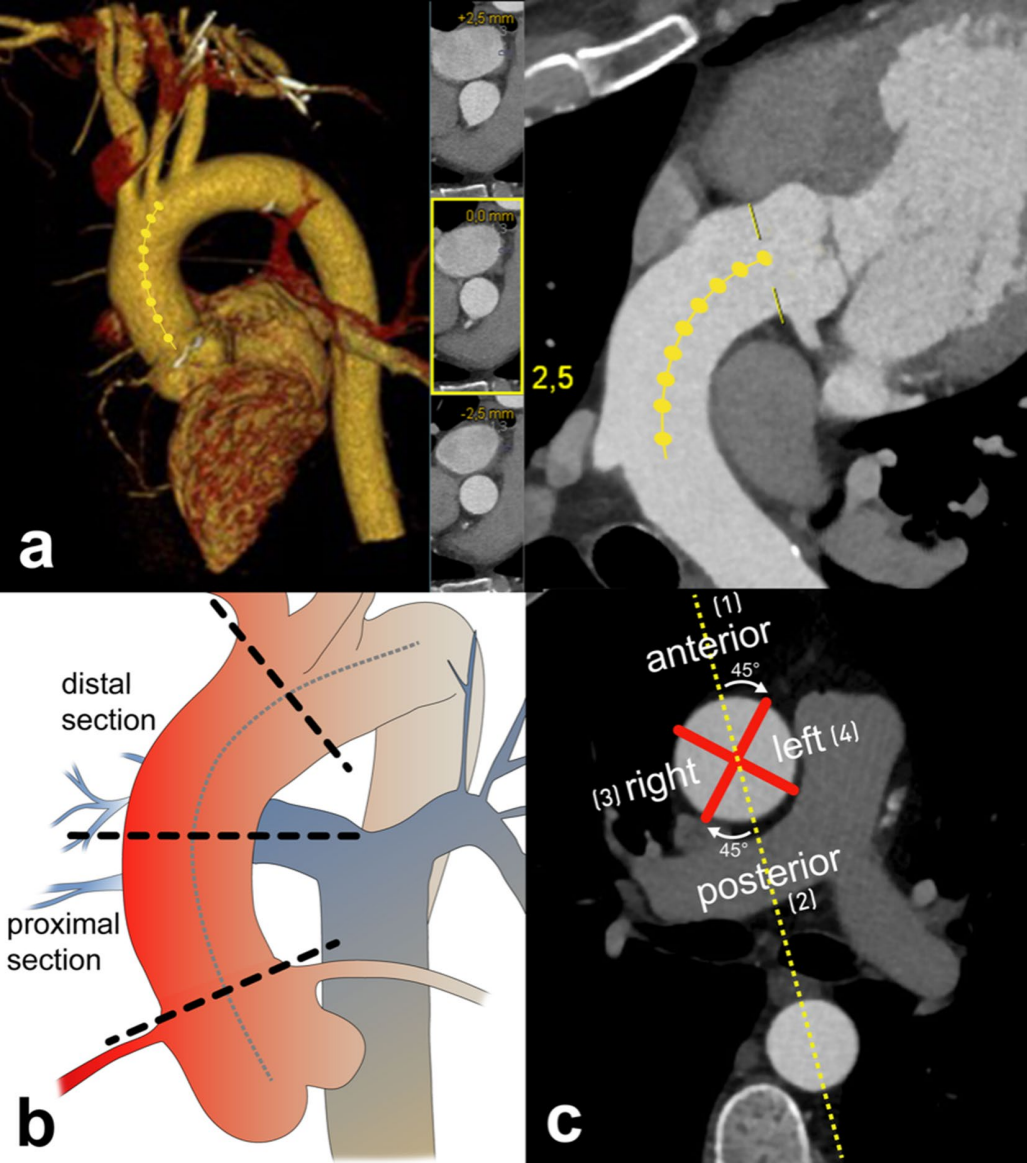
Segmentation of the ascending aorta. “Centerline” of the ascending aorta from the valvular level to the level of the left subclavian artery with the corresponding curved planar reformations (**a**). Allocation of the ascending aorta into a proximal section, extending from the level of the origin of the most distal coronary artery to the level of the origin of the pulmonary artery bifurcation, and a distal section extending from the pulmonary artery bifurcation to the origin of the brachiocephalic trunk (**b**). Sections were determined orthogonally to the course of the “centerline”. For segmentation (**c**), a straight line was drawn through the center of the ascending and corresponding descending aorta at the level of the pulmonary artery bifurcation: (1) the anterior segment covered the sectors 45° on both sides of this straight line in the ventral part of the ascending aorta; (2) the posterior segment included the sectors 45° on both sides in the dorsal part of the ascending aorta. Subsequently, the remaining quarters to the right and to the left of the straight line were termed the (3) right, and the (4) left segment.

The ascending aorta was divided into a defined proximal and distal section that reflected the areas of crossclamping and arterial cannulation during a standard CABG procedure, as described by Johnson et al.^18^. For this purpose, a “centerline” of the ascending aorta was delineated from the valvular level to the level of the left subclavian artery using semi-automatic image processing software (Syngo.Via, Siemens Healthineers, Fig. 1a). The proximal section of the ascending aorta extended from the level of the origin of the most distal coronary artery to the level of the origin of the pulmonary artery bifurcation, determined orthogonally to the course of the “centerline”. Subsequently, the distal section of the ascending aorta extended from the pulmonary artery bifurcation to the origin of the brachiocephalic trunk (Fig. 1b). In addition, both sections were divided into four segments (Fig. 1c).

During a standard CABG procedure, crossclamping affects all segments of the proximal section of the ascending aorta, and cannulation is performed mainly in the distal anterior and left segment. Therefore, these six segments were defined as mechanically stressed segments.

**Figure 2:**
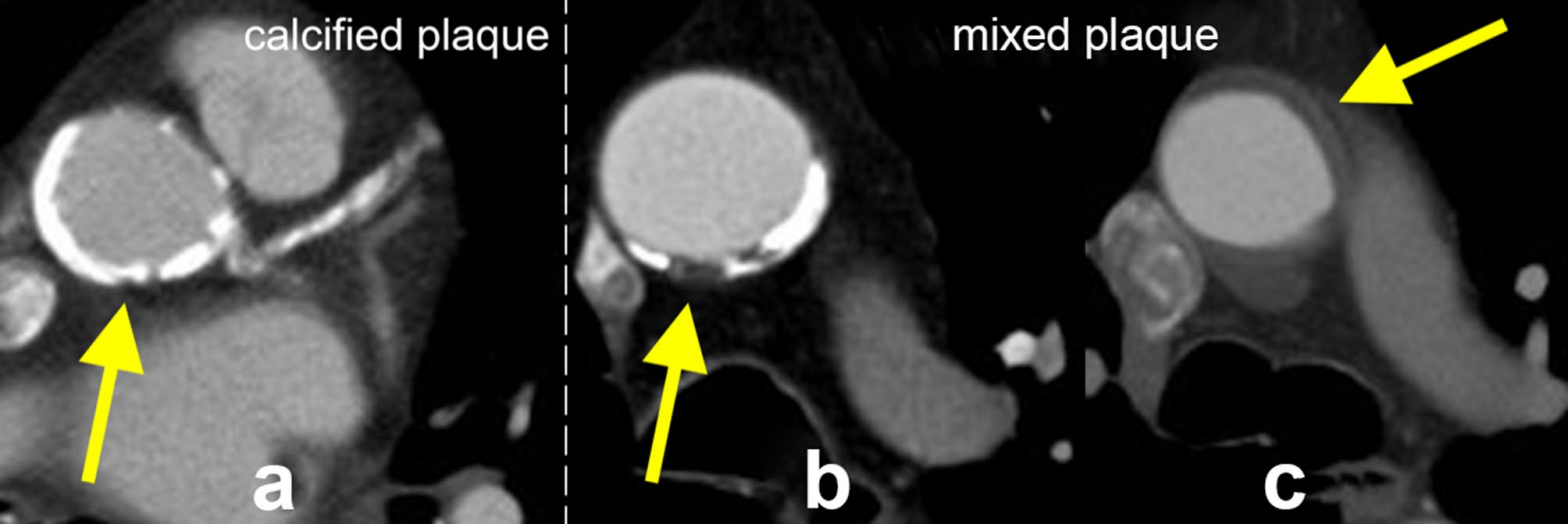
Plaque characterization. Plaque that consisted of exclusively calcified components (HU ≥ 130) was classified as calcified plaque (**a**). Plaques that consisted of calcified (HU ≥ 130) and non-calcified (HU < 130) components (**b**), as well as exclusively non-calcified components (**c**), were graded as mixed plaques.

The presence of atherosclerotic plaque was defined as intimal thickening ≥ 4 mm^19^. Differentiation of atherosclerotic plaques was based on the HU of their components. Components were defined as calcified at a threshold value of ≥ 130 HU, and as non calcified < 130 HU, respectively^20,21^. Subsequently, plaques that consisted of exclusively calcified components were classified as calcified plaques. Due to the known increased risk of rupture of non-calcified plaque components^7,8^, plaques that consisting of any non-calcified components were classified as mixed plaques (Fig. 2a–c).

Based on these classifications, defined segments of the ascending aorta were screened for plaques, which were graded as a dichotomous variable. Image evaluation was performed by two board-certified radiologists (U.A., S.B.P.), with 10 and 15 years of experience in cardiovascular imaging, respectively, by consensus. Extent of disease was defined as the number of concomitantly involved segments.

### Coronary artery bypass grafting and perioperative stroke

Patients referred for CABG underwent careful duplex evaluation of the carotid, vertebral, and subclavian arteries as part of their preoperative vascular assessment. Isolated CABG was performed as a standard procedure as described previously by Johnson et al.^18^. Based on preoperative ungated high-pitch CTA imaging analysis for plaque distribution, modifications were made to the CABG procedure; most frequently this included the use of alternate cannulation sites, beating-heart, or off-pump procedures.

Patients with new-onset neurological disorders after CABG were routinely evaluated by a neurologist in service who ordered computed tomography of the brain at his/her discretion. Consequently, patient records were screened for neurological consultations and concomitant computed tomography scans of the brain to identify patients with stroke. Based on these examinations, stroke was defined according to the STS National Adult Cardiac Surgery Database as any confirmed new neurological deficit of abrupt onset caused by a disturbance in blood supply to the brain that did not resolve within 24 h, and which included additional detection of a new lesion by a computed tomography scan of the brain.

Stroke rates were compared with respect to plaque extent, distribution and morphology.

### Statistics

Discrete variables were described with absolute and relative numbers and by using contingency tables; possible differences in discrete variables between groups were tested with the Chi-Square-test, the Fisher exact test, and the McNemar test as appropriate. Continuous variables were described as mean ± standard deviation, or median and interquartile range (IQRs), as appropriate. Normal distribution of data was tested with the Shapiro–Wilk-Test. Possible differences in continuous variables between groups were tested by the Wilcoxon test, the Kruskal–Wallis test, or the *T *test, as appropriate. All tests were two-sided. No formal Bonferroni correction was applied in this exploratory study. *P* values are given as calculated and should be interpreted with care, considering alpha error accumulation. Results were regarded as statistically significant, if *p* < 0.05. Statistical analyses were performed using SPSS for Windows (version 20.0; IBM Corporation).

## Results

### Plaque burden and distribution

The mean DLP was 515.6 ± 166.8 mGy × cm. Atherosclerotic plaques in the ascending aorta were detected in 159/719 patients (22.1%, Table 3). The proximal section of the ascending aorta was more frequently affected than the distal section (n = 112/719, 15.6% vs. n = 99/719, 13.8% of patients). Overall, the number of patients with calcified plaques (n = 142/719; 19.7%) was significantly higher than those with mixed plaques (n = 34/719; 4.7%, *p* < 0.001). In 133/719 patients (18.5%) at least one plaque was detected in one of the mechanically stressed segments. For these segments, the number of patients with calcified plaques was also significantly higher than those with mixed plaques (n = 114/719, 15.9% vs. n = 26/719, 3.6%, *p* < 0.001).

**Table 3:** Plaque burden per patient.

Patients (n = 719)				
	Any plaque number of patients (%)	Calcified plaque number of patients (%)	Mixed plaque number of patients (%)	*p*
Ascending aorta, overall	159/719 (22.1%)	142/719 (19.7%)	34/719 (4.7%)	< 0.001
Ascending aorta, proximal	112/719 (15.6%)	102/719 (14.2%)	15/719 (2.1%)	< 0.001
Ascending aorta, distal	99/719 (13.8%)	82/719 (11.4%)	24/719 (3.3%)	< 0.001
Mechanically stressed segments	133/719 (18.5%)	114/719 (15.9%)	26/719 (3.6%)	< 0.001

Any plaque, patients with either calcified and/or mixed plaques; *p*, calcified plaque versus mixed plaque.

Overall, atherosclerotic plaques were detected in 336 segments; 164 segments in the proximal section of the ascending aorta and 172 segments in the distal section of the ascending aorta. Atherosclerotic disease affected only one segment of the ascending aorta in 50.3% (n = 80/159) of the patients. Based on atherosclerotic plaque distribution, the most frequently affected segments were the proximal left and the distal posterior segment, in 40.9% (n = 65/159) and 39.0% (n = 62/159) of patients, respectively. Data about the number of involved segments and the distribution of atherosclerotic plaques stratified by plaque composition are presented in Table 4.

**Table 4:** Plaque burden, composition, and distribution in patients with atherosclerotic disease of the ascending aorta.

Patients with atherosclerotic disease in the ascending aorta (n = 159)				
	Any plaque number of patients (%)	Calcified plaque number of patients (%)	Mixed plaque number of patients (%)	*p*
**Plaque distribution proximal section**				
Anterior	53/159 (33.3)	43/159 (27.0)	10/159 (6.3)	< 0.001
Right	18/159 (11.3)	14/159 (8.8)	4/159 (2.5)	
Left	65/159 (40.9)	60/159 (37.7)	5/159 (3.2)	
Posterior	28/159 (17.6)	25/159 (15.7)	3/159 (1.9)	
**Plaque distribution distal section**				
Anterior	36/159 (22.6)	24/159 (15.1)	12/159 (7.5)	< 0.001
Right	27/159 (17.0)	18/159 (11.3)	9/159 (5.7)	
Left	47/159 (29.5)	39/159 (24.5)	8/159 (5.0)	
Posterior	62/159 (39.0)	46/159 (28.9)	16/159 (10.1)	
**Number of affected segments**				
1	80/159 (50.3)	82/159 (51.6)	20/159 (12.6)	< 0.001
2	40/159 (25.2)	29/159 (18.2)	9/159 (5.7)	
3	17/159 (10.7)	17/159 (10.7)	0/159	
4	5/159 (3.2)	3/159 (1.9)	0/159	
5	5/159 (3.2)	4/159 (2.5)	2/159 (1.3)	
6	7/159 (4.4)	5/159 (3.2)	2/159 (1.3)	
7	2/159 (1.3)	0/159	1/159 (0.6)	
8	3/159 (1.9)	2/159 (1.3)	0/159	
Total	= 336 segments	= 336 segments		

Any plaque, patients with either calcified and/or mixed plaques; *p*, calcified versus mixed plaque.

### Perioperative stroke

Of 719 patients studied, 12 patients (1.7%) experienced a perioperative stroke. The incidence of stroke was 7.6% (n = 12/159) in patients with atherosclerotic plaques compared to 0% (n = 0/560) in patients with no plaque (*p* < 0.001). In addition, stroke rates in patients with at least one plaque in mechanically stressed segments were 9.0% (n = 12/133) versus 0% (n = 0/586) in patients without (*p* < 0.001). In addition, we observed a significant association between stroke rate and the number of segments with atherosclerotic plaques (*p* < 0.001). When patients with and without calcified plaques were compared, stroke rates were 3.5% (n = 5/142) versus 1.2% (n = 7/577), which showed no significant difference (*p* = 0.068). Patients with mixed plaques demonstrated significantly higher stroke rates of 20.6% (n = 7/34), compared to 0.7% (n = 5/685) in patients without mixed plaques (*p* < 0.001).

## Discussion

In the present study we have shown that 22.1% (n = 159/719) of patients referred for CABG had atherosclerotic plaques in the ascending aorta, with calcified plaques significantly more frequently detected than mixed plaques. We observed a significantly higher perioperative stroke rate of 7.6% (n = 12/159) in patients with plaque compared to 0% (n = 0/560) in patients with no plaque in the ascending aorta. In addition, we found an association between plaque composition and stroke rate, which was significantly higher in patients with mixed plaques.

Atherosclerotic disease as an important risk factor for patients who are undergoing CABG mainly affects the distal section of the ascending aorta^3,22^, which is line with our findings, where the distal section was most frequently affected in 62.3% (n = 99/159) of patients with atherosclerotic disease.

Although intra-operative epi-aortic ultrasound is the most sensitive modality for the detection of atherosclerotic plaques, it is hampered by the lack of opportunity for preoperative planning^9,23^. In this context, the reference method for imaging of the ascending aorta is conventional CTA, which proved to be superior compared to TEE in plaque detection^24^. The major strength of CTA is the acquisition of three-dimensional, reproducible, surgeon-friendly images of the vascular anatomy^9^. By means of detection and differentiation of composition of plaques, the elimination of pulsation artifacts may be considered to be the key requirement of CTA of the ascending aorta prior CABG. This feature can be achieved with different protocols, including gated non high-pitch, as well as gated or ungated high-pitch scans^12,14,25^. In this context, ungated high-pitch protocols demonstrated excellent image quality at similar or even lower radiation dose compared with both, gated non high-pitch and high-pitch protocols^11,12^. Compared to the use of ECG gating, a further advantage of the ungated high-pitch protocol is time saving, in terms of patient preparation and setting optimal CT parameters^12^.

CABG candidates are frequently suffering from impaired renal function and considered to be at increased risk of contrast-induced nephropathy (CIN)^26^. In this context, reduction of the amount of contrast media has been demonstrated for high-pitch compared to conventional CTA^13^. Although the topic related to the risk of CIN could appear controversial^27^, an optimized scan protocol to reduce any potential risk before CABG, as applied in our study, seems to be mandatory. The feasability and clinical usefullness of a low contrast scan-protocol was demonstrated in several studies focusing on high-pitch CTA prior transcatheter aortic valve implantation^28,29^.

To our knowledge, this is the first study with the use of ungated high-pitch CTA that addresses plaque composition in the ascending aorta prior to CABG. We could demonstrate that a significantly higher number of patients presented with calcified plaques compared to mixed plaques. This may be linked to the high number of patients (75%) treated with lipid-lowering drugs which can lead to an increase of calcifications in atherosclerotic plaques^30^.

Perioperative stroke is a life-threatening complication after CABG, with an incidence of 1.0–3.6%^2,31,32^, which is in line with our results. Stroke risk was found to be dependent on the presence and extent of atherosclerotic disease, with an incidence of 8.7% among patients with atherosclerotic plaques^22^, which is in line with the stroke rate of 7.6% (n = 12/159) in our study. Given the general assumption that perioperative strokes result mainly from cerebral embolization of atheromatous debris and not from calcified material^22^, we could demonstrate a significantly higher stroke rate in patients with mixed plaques.

During cannulation and aortic clamping mechanical manipulation in affected regions poses a risk for detachment of especially non-calcified plaque components and subsequent embolization^33,34^. This mechanism is supported by our significantly higher stroke rate in patients with at least one atherosclerotic plaque in a mechanical stressed segment compared to patients without. In addition, our findings underline the importance of preoperative ungated high-pitch CTA imaging to optimize cannulation and aortic clamping strategies. In this context, several studies could demonstrate a reduction in stroke rates through adequate surgical planning and adaptation of the standard surgical CABG procedure to reduce or avoid mechanical manipulation^10,33,34^.

Beside the established vascular risk factors for stroke such as hypertension, dyslipidemia, smoking history, or diabetes mellitus, patients undergoing CABG are additionally burdened by secondary well-known risk factors like prosthetic cardiac valves, low ejection fraction, atrial fibrillation, and carotid stenosis^35^. Comparing patients with and without perioperative stroke by means of these variables, our data did not demonstrate significant differences (Table 1).

The major limitation of our study was its retrospective design. Second, the outcome variable was a clinically evident neurologic deficit confirmed by computed tomography of the brain. No advanced neurocognitive testing was performed that would have allowed for detection of subtle differences in cognitive impairment. Given the fact, that diffusion weighted magnetic resonance imaging is the gold-standard to detect small, transient ischemic lesions^36^, computed tomography-negative, minor neurologic deficits could have been missed. Third, surgeons performed modifications of the surgical strategy at their discretion. The low event rates did not permit a meaningful comparison of stroke rates in patients who did or did not undergo modifications of the CABG procedure. Fourth, the low event rate significantly restricts the use of a multivariable model. Therefore, we are not able to demonstrate a relationship of further variables (e.g. sex, age, dyslipidemia, smoking history, diabetes mellitus, carotid stenosis or history of stroke) with perioperative stroke. Fifth, since eight patients who suffered from stroke presented with atherosclerotic plaques in more than one segment, our data do not allow a statement with respect to the causative diseased segment.

In summary, all patients with perioperative stroke presented with atherosclerotic disease of the ascending aorta. The stroke rate was significantly associated with the presence and extent of atherosclerotic disease. Patients burdened with mixed plaques presented with significantly higher perioperative stroke rates. To our knowledge, this is the first study to demonstrate that detection of plaque extent and composition in the ascending aorta may be helpful to improve risk stratification of stroke in patients prior to CABG. Further prospective studies, evaluating the relationship of atherosclerosis in the ascending aorta and minor neurological deficits related to CABG are warranted.

## Data Availability

The dataset generated during the current study are available from the corresponding author on reasonable request.

## Abbreviations

CTA: Computed tomography angiography
CABG: Coronary artery bypass grafting
TEE: Transesophageal echocardiography
TTE: Transthoracic echocardiography
HU: Hounsfield unit
ECMO: Extracorporeal membrane oxygenation

## References

[1] Stamou SC (2001). Stroke after coronary artery bypass: incidence, predictors, and clinical outcome. Stroke.

[2] Tarakji KG, Sabik JF, Bhudia SK, Batizy LH, Blackstone EH (2011). Temporal onset, risk factors, and outcomes associated with stroke after coronary artery bypass grafting. JAMA.

[3] Dávila-Román VG (1994). Atherosclerosis of the ascending aorta. Prevalence and role as an independent predictor of cerebrovascular events in cardiac patients. Stroke.

[4] Hogue CW, Murphy SF, Schechtman KB, Dávila-Román VG (1999). Risk factors for early or delayed stroke after cardiac surgery. Circulation.

[5] Amarenco P (1992). The prevalence of ulcerated plaques in the aortic arch in patients with stroke. N. Engl. J. Med..

[6] Kapetanakis EI (2004). The impact of aortic manipulation on neurologic outcomes after coronary artery bypass surgery: a risk-adjusted study. Ann. Thorac. Surg..

[7] Virmani R, Burke AP, Farb A, Kolodgie FD (2006). Pathology of the vulnerable plaque. J. Am. Coll. Cardiol..

[8] Mann JM, Davies MJ (1996). Vulnerable plaque. Relation of characteristics to degree of stenosis in human coronary arteries. Circulation.

[9] Park K-H (2010). Clinical impact of computerised tomographic angiography performed for preoperative evaluation before coronary artery bypass grafting. Eur. J. Cardiothorac. Surg..

[10] Nakamura M (2008). Does intensive management of cerebral hemodynamics and atheromatous aorta reduce stroke after coronary artery surgery?. Ann. Thorac. Surg..

[11] Karlo C (2011). High-pitch dual-source CT angiography of the aortic valve-aortic root complex without ECG-synchronization. Eur. Radiol..

[12] Beeres M (2012). High-pitch dual-source CT angiography of the whole aorta without ECG synchronisation: initial experience. Eur. Radiol..

[13] Apfaltrer P (2012). Radiation dose and image quality at high-pitch CT angiography of the aorta: intraindividual and interindividual comparisons with conventional CT angiography. AJR Am. J. Roentgenol..

[14] Wielandner A (2016). Is ECG triggering for motion artefact reduction in dual-source CT angiography of the ascending aorta still required with high-pitch scanning? The role of ECG-gating in high-pitch dual-source CT of the ascending aorta. Br. J. Radiol..

[15] Nakajima S (2017). Clinical application of effective atomic number for classifying non-calcified coronary plaques by dual-energy computed tomography. Atherosclerosis.

[16] Schuhbäck A (2012). Interobserver agreement for the detection of atherosclerotic plaque in coronary CT angiography: comparison of two low-dose image acquisition protocols with standard retrospectively ECG-gated reconstruction. Eur. Radiol..

[17] Sandner SE (2020). Routine preoperative aortic computed tomography angiography is associated with reduced risk of stroke in coronary artery bypass grafting: a propensity-matched analysis. Eur. J. Cardiothorac. Surg..

[18] Johnson WD, Flemma RJ, Lepley D, Ellison EH (1969). Extended treatment of severe coronary artery disease: a total surgical approach. Ann. Surg..

[19] Kronzon I, Tunick PA (2006). Aortic atherosclerotic disease and stroke. Circulation.

[20] Agatston AS (1990). Quantification of coronary artery calcium using ultrafast computed tomography. J. Am. Coll. Cardiol..

[21] Szilveszter B, Celeng C, Maurovich-Horvat P (2016). Plaque assessment by coronary CT. Int. J. Cardiovasc. Imaging.

[22] van der Linden J, Hadjinikolaou L, Bergman P, Lindblom D (2001). Postoperative stroke in cardiac surgery is related to the location and extent of atherosclerotic disease in the ascending aorta. J. Am. Coll. Cardiol..

[23] Rosenberger P (2008). The influence of epiaortic ultrasonography on intraoperative surgical management in 6051 cardiac surgical patients. Ann. Thorac. Surg..

[24] Chatzikonstantinou A (2012). CT angiography of the aorta is superior to transesophageal echocardiography for determining stroke subtypes in patients with cryptogenic ischemic stroke. Cerebrovasc. Dis..

[25] Schernthaner RE (2012). Dose modulated retrospective ECG-gated versus non-gated 64-row CT angiography of the aorta at the same radiation dose: comparison of motion artifacts, diagnostic confidence and signal-to-noise-ratios. Eur. J. Radiol..

[26] Cooper WA (2006). Impact of renal dysfunction on outcomes of coronary artery bypass surgery: results from the Society of Thoracic Surgeons National Adult Cardiac Database. Circulation.

[27] McDonald RJ (2013). Intravenous contrast material-induced nephropathy: causal or coincident phenomenon?. Radiology.

[28] Bittner DO (2016). Contrast volume reduction using third generation dual source computed tomography for the evaluation of patients prior to transcatheter aortic valve implantation. Eur. Radiol..

[29] Dankerl P (2017). Computer-aided evaluation of low-dose and low-contrast agent third-generation dual-source CT angiography prior to transcatheter aortic valve implantation (TAVI). Int. J. Comput. Assist. Radiol. Surg..

[30] Nakazato R (2012). Statins use and coronary artery plaque composition: results from the International Multicenter CONFIRM Registry. Atherosclerosis.

[31] Roach GW (1996). Adverse cerebral outcomes after coronary bypass surgery. Multicenter study of perioperative ischemia research group and the ischemia research and education foundation investigators. N. Engl. J. Med..

[32] Filsoufi F, Rahmanian PB, Castillo JG, Bronster D, Adams DH (2008). Incidence, topography, predictors and long-term survival after stroke in patients undergoing coronary artery bypass grafting. Ann. Thorac. Surg..

[33] Edelman JJ, Yan TD, Bannon PG, Wilson MK, Vallely MP (2011). Coronary artery bypass grafting with and without manipulation of the ascending aorta: a meta-analysis. Heart Lung Circ..

[34] Vallely MP (2008). Anaortic techniques reduce neurological morbidity after off-pump coronary artery bypass surgery. Heart Lung Circ..

[35] Esenwa C, Gutierrez J (2015). Secondary stroke prevention: challenges and solutions. Vasc. Health Risk Manag..

[36] Gass A, Ay H, Szabo K, Koroshetz WJ (2004). Diffusion-weighted MRI for the ‘small stuff’: the details of acute cerebral ischaemia. Lancet Neurol..

